# Evaluation of Anti-oxidant and Anti-biofilm Activities of Biogenic Surfactants Derived from *Bacillus amyloliquefaciens* and *Pseudomonas aeruginosa*

**DOI:** 10.22037/IJPR.2020.1101033

**Published:** 2020

**Authors:** Soosan Abdollahi, Zahra Tofighi, Tahereh Babaee, Mohammad Shamsi, Ghazal Rahimzadeh, Hossein Rezvanifar, Elaheh Saeidi, Morteza Mohajeri Amiri, Yasaman Saffari Ashtiani, Nasrin Samadi

**Affiliations:** a *Department of Drug and Food Control, Faculty of Pharmacy, Tehran University of Medical Sciences, Tehran, Iran. *; b *Department of Pharmacognosy, Faculty of Pharmacy, Tehran University of Medical Sciences, Tehran, Iran. *; c *Department of Food Science and Technology, Science and Research Branch, Islamic Azad University, Tehran, Iran.*; d *Department of Drug and Food Control, Faculty of Pharmacy, International Campus, Tehran University of Medical Sciences, Tehran, Iran. *; e *Pharmaceutical Quality Assurance Research Center, The Institute of Pharmaceutical Sciences (TIPS), Tehran University of Medical Sciences, Tehran, Iran.*

**Keywords:** Biosurfactant, Antioxidant activity, Biofilm, Surfactin, Rhamnolipid

## Abstract

Biosurfactants, the microbial originated surface active agents, can modify the physicochemical properties of surfaces and reduce the bacterial adhesion via changing bacterial adhesion interactions on surfaces. They were also able to block oxidative chain reactions and might show antioxidant properties. The goal of this study was to evaluate the antioxidant and antibiofilm activities of biosurfactants which were derived from two autochthonous biosurfactant-producing strains,* Bacillus amyloliquefaciens *NS6 (surfactin), and *Pseudomonas aeruginosa *MN1 (rhamnolipids). Their antioxidant activities were determined by ferric reducing antioxidant power (FRAP) and 1,1-diphenyl-2-picrylhydrazyl (DPPH) methods. Ferric thiocyanate (FTC) assay was used for determination of their lipid peroxidation inhibition capacity. Their effect to reduce the adhesion of *Streptococcus mutans* on polystyrene surfaces and disruption of its pre-formed biofilms were also investigated. Our results indicated that surfactin showed higher antioxidant activity than rhamnolipids and showed relatively similar efficiency to BHA that suggests it as a good alternative for synthetic antioxidants. In other hand, rhamnolipid conditioned surfaces showed higher antiadhesive and antibiofilm activity in comparison with surfactin treated surfaces.

## Introduction

Biosurfactants are bioactive substances that are mainly produced by microorganisms in hydrophobic media. Due to intrinsic surface activity and some particular properties of biosurfactants like low toxicity, diversity, acceptable activity at extreme conditions, ability of large scale production, environmental biocompatibility and biodegradability, variety of applications have been raised for them in food, petroleum, and pharmaceutical industries (1-6). Basically there are six major types of biosurfactants: hydroxylated and cross linked fatty acids (mycolic acids), glycolipids, lipopolysaccharides, lipoproteins-lipopeptides, phospholipids, and the complete cell surface itself (7).

Recently it has been illuminated that some of biosurfactants are effective antioxidants promising to be alternative or even complete replacement for synthetic antioxidants (8, 9). Antioxidants are the substances which can act as free radical scavengers and terminate both *in-vivo* and* in-vitro *oxidation chain reactions by providing hydrogen groups to free radicals or playing their role as electron donors (10). In pharmaceutical, cosmetic and food industries, antioxidants are considered as essential additives that are used for preservation of different products by hindering oxidative rancidity of lipids and retarding their spoilage (4, 11). According to the carcinogenic and toxic effects of synthetic antioxidants, there is an incremental interest for substituting antioxidants from various natural sources instead of synthetics (12-16). 

Biofilm refers to a surface adherent, multi species, association of microorganisms in an extracellular polymeric substance matrix, called EPSs (17). Caries, gingivitis, periodontitis, endocarditis, and prostatitis are some well-known biofilm associated diseases added to industrial and environmental problems caused by biofilms (18-22). Biofilms are usually resistant to current antibiotics and disinfectants due to their specific growth condition and resistant gene transformation. Biosurfactants seems to be great natural alternative compounds for chemical anti-biofilms due to their surface activity and antimicrobial properties (23, 24).

In the present study, glycolipid biosurfactant (rhamnolipids) and lipopeptide biosurfactant (surfactin) were produced by *Pseudomonas aeruginosa* MN1 and *Bacillus amyloliquefaciens* NS6, respectively. Antioxidant activities of these biosurfactants were evaluated by three different methods. Among various methods established for evaluation of antioxidant efficacy, DPPH method was selected as a routine straightforward method to determine free radical scavenging capacity of biosurfactants. FRAP method that evaluates total antioxidant activity, was used as general method for measuring the reducing power of antioxidants. Also, FTC method that demonstrates the ability of antioxidants in preventing lipid peroxidation was used to assess whether these biosurfactants were appropriate to be used as preservative in high-fat products. Along with biosurfactants, common natural and synthetic antioxidants such as vitamin C, vitamin E, and BHA were examined concomitantly as positive controls. Due to the intrinsic surface activity of biosurfactants and their potential use in the control of biofilm formation, the antibacterial, antibiofilm, and antiadhesive activities of biosurfactants derived from *P. aeruginosa* MN1 and *B. amyloliquefaciens *NS6 against *Streptococcus mutans*, as one of the major etiological agent in dental caries, were also examined. 

## Experimental

*Microorganisms and biosurfactants isolation*

*Pseudomonas aeruginosa *(*P. aeruginosa*) MN1 and *Bacillus amyloliquefaciens *(*B. amyloliquefaciens*) NS6 with GenBank accession number of KF177175 were previously isolated from oil contaminated soil (25). After cultivation on nutrient agar (NA; Merck Co., Darmstadt, Germany) plates and incubation at 35 °C for 24 h, bacterial colonies were individually transferred to Erlenmeyer flasks containing 100 mL of nutrient broth medium (NB; Merck Co., Darmstadt, Germany) and incubated at 35 °C for 24 h. Then, 10 mL of each culture broth was individually transferred to 500-mL Erlenmeyer flask containing 100 mL of Mineral Salt Medium (MSM) that was prepared according to the method described by Linhardt *et al.* (26).

The MSM medium contained (g/L) NaNO_3_, 15; KCl, 1.1; NaCl, 1.1; FeSO_4_.7H2O, 0.00028; KH_2_PO_4_, 4.3; K_2_HPO_4_, 3.4; MgSO_4_.7H_2_O, 0.5; yeast extract 0.5; and 5 mL of a trace element solution. The trace element solution contained (g/L): ZnSO_4_.7H_2_O, 0.29; CaCl_2_.4H_2_O, 0.24; CuSO_4._5H_2_O, 0.25; and MnSO_4_ H_2_O, 0.17. The trace element solution was filter sterilized through a 0.22-μm membrane filter and was then added to the medium. Finally, 50 g/L of glycerol was added to the medium as a carbon source. Erlenmeyer flasks were placed in a shaker incubator at 30 °C and 150 rpm for 24 h. For isolation of the biosurfactant from culture medium, centrifugation at 8000 ×g and 4 °C for 20 min was done to eliminate the cell bodies. The collected supernatants were acidified to pH 1.0-2.0 and placed overnight at 4 °C. Then the supernatants were fractionated by addition of organic solvents as chloroform/methanol 2:1 (v/v) at 4 °C for at least twice. Organic phases that contained biosurfactants were collected and concentrated by a rotary evaporator (Büchi, Switzerland). The concentrate was analysed by thin-layer chromatography (TLC) on silica gel (K6F 60 A, Whatman, Inc.) with a solvent system of chloroform, methanol, and acetic acid (65:15:2 v/v/v). The TLCs were visualized using ninhydrin reagent in order to indicate peptides or α-naphthol-sulphuric acid to stain glycolipids.

*Structural characterization of the biosurfactant*

Chemical composition of the rhamnolipid biosurfactant was previously characterized by liquid chromatography-tandem mass spectrometry (LC-MS/MS) analysis (25). The isolated biosurfactant from *B. amyloliquefaciens* NS6 was characterized by ^1^H-NMR. The compound was dissolved in CDCl_3_ and the spectra was recorded at room temperature using Bruker DRX (Germany) spectrometer 500 MHZ. Chemical shifts (δ) are given on the ppm scale relative to tetramethylsilane (TMS).

*Ferric Reducing Antioxidant Power (FRAP) assay*

The reducing power of biosurfactants was determined by FRAP assay method (27, 28). According to this method, the reduction of ferric to ferrous was performed in the presence of an antioxidant. Then, the ferrous ion conjugated with the ferric cyanide ion to form a prussian blue coloured product. Briefly, 0.1 mL of different concentrations of biosurfactants in purified water was mixed with 0.9 mL ethanol and 5 mL purified water. Then 1.5 mL of hydrochloric acid 1 M, 1.5 mL of potassium ferricyanide 0.2% (w/v), 0.5 ml of sodium dodecyl sulphate (SDS) 1% (w/v), and finally 0.5 mL of ferric chloride 0.1% (w/v) were added to the mixture. After heating the mixture in a 50 °C water bath for 20 min, the samples were rapidly cooled on ice. Then, their absorbance was determined at 700 nm by a spectrophotometer (Cecil-9200, UK). Total antioxidant activities of the samples and vitamin C (as positive control) were measured in comparison with the standard solutions of FeSO_4_. 

*DPPH (1-diphenyl-2-picrylhydrazyl) assay*

In this method, the antioxidant activities of the biosurfactants were determined on the basis of their scavenging activities of 1, 1-diphenyl-2-picrylhydrazyl (DPPH) free radicals according to Goodarzi *et al.* (29). Briefly, 1 mL aliquots of different concentrations of biosurfactants in methanol were added to 2 mL of DPPH methanolic solution (40 μg/mL). The mixtures were shaken vigorously and left for 30 min at room temperature in dark. Then, absorbance of the solutions was determined at 517 nm by a spectrophotometer. The same procedures were used for methanol as blank, BHA, and vitamin E as positive controls. The antioxidant activities were then calculated as the percentage of inhibition according to the following Equation:

AA% = [1 - ((A_sample_ – A_control_)/A_blank_)] × 100

Where, A _blank_ is the absorbance of the blank, A _sample_ is the absorbance of the biosurfactant, and A _control_ is the absorbance of the negative control (maximum concentration of samples without DPPH). 

Antioxidant activity of each sample was then expressed as the half maximal inhibitory concentration (IC_50_), which denotes the concentration of sample required for scavenging 50% of the DPPH free radicals or the concentration of the sample which led to 50% decrease of the initial DPPH concentration.

*Ferric Thiocyanate assay (FTC)*

According to the method of Yassa *et al. *(30), 1 mL of different concentrations of biosurfactants in methanol were added to 1 mL of sodium phosphate buffer (0.05 M, pH 7.0) and 0.5 mL of 2.5% (v/v) linoleic acid emulsion that was prepared by mixing of linoleic acid and tween 20 in methanol. The mixtures were incubated at 40 °C for 96 h. Then, aliquots of 0.1 mL of these mixtures were taken at 24 h time intervals and mixed with 4.7 mL of ethanol (70%, v/v), 0.05 mL of aqueous ammonium thiocyanate (30%, w/v), and 0.05 mL of ferrous chloride (0.02 M in 3.5% hydrochloric acid) solutions. After 3 min, the absorbance of each mixture was measured at 500 nm by a spectrophotometer. The experiments were repeated every 24 h until one day after that the absorbance of the negative control reached to the maximum (4 days). Ethanol (70%, v/v) as blank, the mixture without biosurfactant as negative control, and vitamin E and BHA as positive controls were examined in the same manner as described above. 

The oxidation index (OI) of the samples and negative control at each time interval were defined as: 

OI = Absorbance(t)/Absorbance(t_0_)

Where Absorbance (t) is the absorbance of sample or blank at each time interval and Absorbance (t_0_) is the absorbance of sample or blank at zero time.

These calculated OIs were used for determination of antioxidant activities (AA) of the samples at each time interval by the following Equation: 

Antioxidant activity % = 100 - [OI of sample/OI of negative control × 100]

*Antimicrobial activity*

The antibacterial effect of lipopeptide and glycolipid biosurfactants against *Streptococcus mutans* (*S. mutans*) ATCC 35668 was assessed by microdilution method for planktonic cells (31). Separately, 200 µL aliquot of the stock solution of surfactin (160 mg/mL) and rhamnolipid (25 mg/mL) in brain heart infusion medium (BHI, Merck Co. Germany) were added to the first well of each row in 96-well plastic plate and serially diluted in each horizontal row with 100 µL of BHI. Then, 100 µL of bacterial suspension (1 × 10^6^ CFU/mL) was transferred to each well, to reach the final concentration of about 5 × 10^5^ CFU/well. The biosurfactant free medium containing bacterial suspension and the sterile medium were used as non-treated control and blank, respectively. Following 24 h incubation at 35 °C, the wells were tested for the presence of visible bacterial growth in comparison with the control wells. The lowest biosurfactant concentration in which the test bacteria did not show any visible growth was considered as minimum inhibitory concentration (MIC).

*Antiadhesion assay*

The anti-adhesion effect of two biosurfactants against *S. mutans* was determined by using 96-well microtiter plates (32). Separately, a 200 µL aliquot of the stock solution of surfactin (80 mg/mL) or rhamnolipid (12.5 mg/mL) in BHI broth were added to the first well of each row in 96-well microtiter plates, while the other wells contained 100 µL of BHI broth. Then, surfactin and rhamnolipid solutions were serially diluted in each horizontal row to reach 0.63 and 0.024 mg/mL, respectively. Then, 100 µL of bacterial suspension (1 × 10^6^ CFU/mL) was transferred to each well to reach the final concentration of about 5 × 10^5^ CFU/well. Sterile medium was used as negative control. After 24 h of incubation at 37 °C, unattached cells were removed and crystal violet assay was performed as follows. Microtiter wells were filled with 200 µL of methanol (99% purity) to fix the adherent bacteria for 15 min. Then, the wells were emptied, left to dried and filled with 200 µL of crystal violet 2% solution in water for 5 min. Excess stain rinsed out with distilled water stream and the plates were dried at room temperature. The bounded dye was solubilized with 200 µL of 30% acetic acid in water. The absorbance of each well was measured in a microtiter reader at 600 nm, while the 30% acetic acid solution was the blank and the medium without the biosurfactant was used as non-treated control. All the experiments were repeated at least three times. The percentage of cell adhesion compared to biosurfactant free wells was calculated as:

Percentage inhibition = [1 − (A sample/A control)] × 100] 

*Biosurfactant-mediated disruption of pre-formed biofilm*

The potential of extracted biosurfactants to inhibit *S. mutans* biofilm production was evaluated in 96-well plastic microtiter plate (32). The experiments were run in 4-8 replications for each treatment. *S. mutans* was grown in tryptic soy broth (Merck Co., Germany) overnight, and then diluted in BHI broth supplemented with 2% sucrose (BHIS) with 1:100 ratios. Then, 200 µL aliquots of the prepared bacterial suspension were dispensed in wells of 96-well microtiter plate and incubated at 37 °C for 24 h. After that the planktonic cells were discarded from the wells and the medium was replaced by BHIS containing a range of surfactin concentrations from 1.25 to 80 mg/mL or rhamnolipid concentrations- from 0.19 to 12.5 mg/mL. The medium without biosurfactant was used as untreated control. Following further incubation of plates (30 °C, 24 h), the unattached cells were removed and the biofilm plates were washed by phosphate buffered saline (PBS). Crystal violet assay method was used to quantify the biofilm formation as mentioned above. The results were expressed in terms of removal percentage at different biosurfactant concentrations**.**

*Statistical analysis*

The results were expressed as the mean of at least three independent replicates. The means were compared pairwise using Sigmaplot 12.0 statistical software. The Tukey’s test was used to analyse significance between independent groups. The significance was accepted as *P* < 0.05.

## Results and Discussion

*Chemical composition of the isolated biosurfactants*

In our previous study, it was demonstrated that biosurfactant obtained from *P. aeruginosa* MN1 had rhamnolipid structure and contained 16 rhamnolipid homologues. Di-lipid rhamnolipids containing C (10)-C (10) moieties were the most predominant congeners among mono-rhamnose (53.29%) and di-rhamnose (23.52%) homologues. The biosurfactant had the molecular weight of about 548.71 Da (25). 

The ^1^H-NMR spectrum of the biosurfactant from* B. amyloliquefaciens* NS6 showed three main regions which correspond to resonance of amide protons, α-carbon protons, and side-chain protons. The spectrum confirms the presence of a long aliphatic chain (CH_2_ at 1.22–1.26 ppm) and a peptide backbone (NH at 7.27 ppm and CH at 4.2 ppm). NMR analysis indicated the presence of aliphatic hydrocarbons combined with a peptide moiety which confirmed lipopeptide nature of the isolated biosurfactant. This lipopeptide was identified as surfactin (molecular weight of 1036.34 Da), due to its similar spectrogram with standard surfactin (Sigma Co.).

*Ferric Reducing Antioxidant Power (FRAP) assay*

The results of reducing power of different concentrations of surfactin and rhamnolipid biosurfactants in comparison with vitamin C (as positive control) were depicted in Table 1. The results of FRAP assay were expressed as μM FeSO_4_ equivalent that was calculated from FeSO_4 _standard curve (y = 0.0005x + 0.067).

The reducing capacity of 4.5 mM surfactin (255.2 μM FeSO_4_) was comparable with 0.7 mM vitamin C (235.2 μM FeSO_4_). While the reducing power of 9 mM rhamnolipids (537.2 μM FeSO_4_) was comparable with 1.4 mM vitamin C (553.2 μM FeSO_4_). Moreover, the equivalency of the results to FeSO_4_ indicated that surfactin reducing capacity in terms of mM was about 2 times more than that of rhamnolipids. Also, increases in biosurfactants concentrations were associated with increase in their reducing power which represented their dose-dependent pattern. Obviously, both biosurfactants had lower reducing power activities than vitamin C. Yalçin and Ҫavuşoǧlu demonstrated that the reduction potency of lipopeptide biosurfactants may be related to the presence of hydroxyl groups in their molecular structure (33), hence, the lower reducing capacity of rhamnolipids can be attributed to its lower content of hydroxyl groups. Moreover, the hydrophobic amino acids (valine and leucine), acidic amino acids (aspartic acid and glutamic acid) and sulphur-containing amino acids such as methionine enhanced reducing power of surfactin derived from *B. amyloliquefaciens* (34). 

*DPPH (1-diphenyl-2-picrylhydrazyl) assay*

The DPPH assay has been used to investigate the scavenging or proton donating ability of compounds (35). The results of DPPH assay obtained for different concentrations of surfactin (0.45-3.6 mM), rhamnolipids (0.9-7.2 mM), vitamin E (0.025-0.1 mM), and BHA (1-5 mM) were used to find linear regression lines of DPPH activities *vs.* concentrations of these compounds. Then, their IC_50 _values were calculated from the estimated regression lines. As displayed in Table 2, the IC_50_ values of surfactin and rhamnolipids were 2.73 mM and 4.15 mM, respectively. In comparison with vitamin E (IC_50_ of 0.036 mM), both biosurfactants have shown lower antioxidant activity. But the antioxidant activity of BHA (IC_50_ of 2.86 mM) was comparable with surfactin and was higher than rhamnolipids. Jemil *et al.* reported IC_50_ of 357 μg/mL for DCS1 lipopeptide biosurfactants produced by *B. methylotrophicus* which was lower than that obtained for BHA (36). Ben Ayed *et al.* reported that A21 lipopeptide which was produced by *Bacillus mojavensis *showed lower scavenging activity compared with BHA via DPPH assay (35). In the present study, the results of DPPH assay for rhamnolipids and surfactin were dose-dependent. The antioxidant activity of rhamnolipids or surfactin were due to the neutralization of free radicals by transferring protons or electrons (37). The powerful DPPH scavenging activity and also reducing power of surfactin could be explained by the presence of some active residues in the peptide ring including tyrosine residue via its phenolic hydroxyl group and proline residue from its pyrrolidine ring. It was reported that hydrocarbon fatty acid chain enhanced radical scavenging activity, but it was not affected by their chain length diversity. Therefore, biosurfactants with low molecular mass peptide moieties had higher DPPH radical scavenging activities (34). 

*Ferric Thiocyanate (FTC) assay*

**Figure 1:**
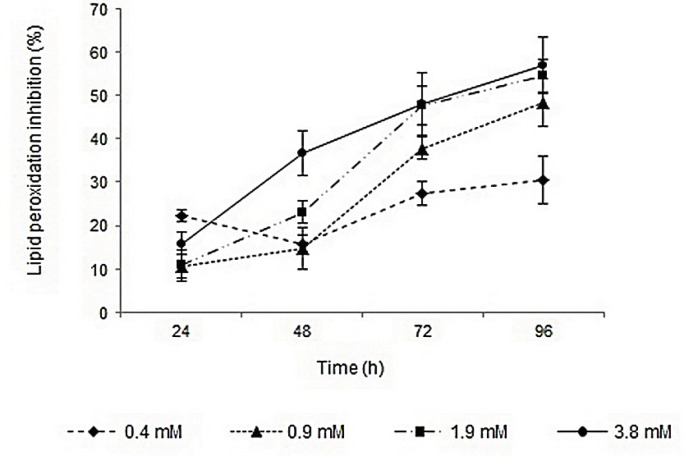
Profile of lipid peroxidation inhibition activity of different concentrations of surfactin during 96 h

**Figure 2:**
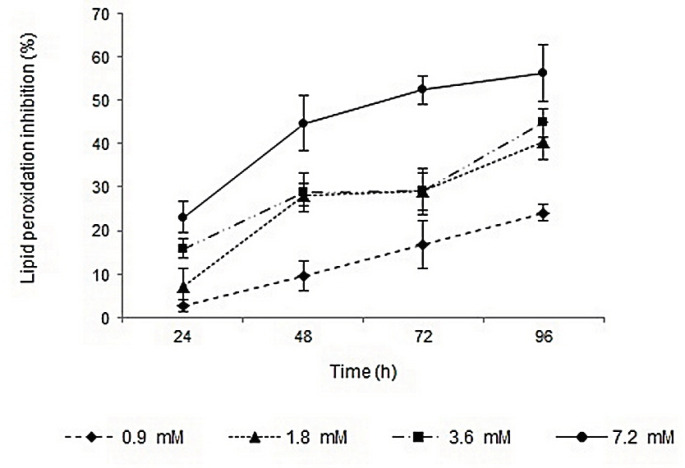
Profile of lipid peroxidation inhibition of different concentrations of rhamnolipids during 96 h

Lipid peroxidation inhibition activities of surfactin, rhamnolipids, vitamin E, and BHA were determined by FTC method. The IC_50_ values of the compounds were calculated based on the regression lines plotted by antioxidant activities *vs.* natural logarithm of their concentrations. Surfactin and rhamnolipids with IC_50_ values of 1.65 and 4.6 mM showed lower activities than vitamin E (IC_50_ value of 0.04 mM). The lipid peroxidation inhibition activities of surfactin and BHA were relatively similar, but rhamnolipids showed lower capacity. Profiles of lipid peroxidation inhibition of two biosurfactants during 96 h were shown in Figures 1 and 2. The inhibition activities were increased by increasing biosurfactants concentrations. Although, in the first 24 h the lipid peroxidation inhibition activities in minimum and maximum concentrations of biosurfactants were close to each other, more differences became apparent after 96 h. Ben Ayed *et al.* reported that A21 lipopeptide lipid peroxidation activity was significantly lower than alpha-tocopherol after 3 days, but after 7 days of incubation and in concentrations of ≥10 mg/mL their lipid peroxidation activity were almost comparable (35). 

In this study, correlation of the data obtained from DPPH and FTC methods were investigated by linear regression analysis. As shown in Table 3, regression equations were extracted from the plot of radical scavenging activities of different concentrations of biosurfactants *vs.* their lipid peroxidation inhibition activities. The positive slope of regression lines indicated perfectly positive correlations between DPPH and FTC results. It means that radical scavenging activity and lipid peroxidation inhibition capacity of two biosurfactants altered by the same pattern and same functional groups were responsible for antioxidant activities in DPPH and FTC methods. High correlation coefficients represented consistency of association between the results in different biosurfactants concentrations.

The inhibitory effect of lipid peroxidation of the surfactin could be explained by the presence of hydrophobic amino acids in the peptide ring and acyl chain of beta-hydroxy fatty acids which improve solubility of the peptide in hydrophobic media (34). 

The DPPH scavenging activities and also lipid peroxidation inhibition capacities were influenced by increasing the number of double bonds in fatty acid chains. It was demonstrated that unsaturated lipids were able to scavenge reactive oxygen species and prevent reactions of lipid peroxidation. Therefore, glycolipid biosurfactants with unsaturated fatty acids were really powerful antioxidants (9). In our study, the low antioxidant activity of glycolipid biosurfactant derived from *P. aeruginosa* MN1 could be attributed to lower content of unsaturated fatty acids (5.9% of rhamnolipid homologues) (25). 

*Antibacterial activity*

The antibacterial activities of surfactin and rhamnolipid biosurfactants against *S. mutans* planktonic cells were evaluated by broth microdilution method. Partially purified rhamnolipids showed higher antibacterial activity and inhibited *S. mutans* growth at 6.25 mg/mL. The MIC of lipopeptide biosurfactant against *S. mutans* was about 50 mg/mL. 

*Antiadhesive effect*

The ability of a series of biosurfactants concentrations to reduce the adhesion of *S. mutans *to polystyrene surfaces, after preconditioning of the surface with biosurfactants, was evaluated by 96-well microtiter plates. The *S. mutans* adhesion was reduced by 93.7% with 12.5 mg/mL of rhamnolipid, whereas similar reduction (94.8%) in bacterial adhesion was observed in the surfaces conditioned with 80 mg/mL of surfactin (Tables 4 and 5). The statistical comparison indicated that there were significant differences (*P* < 0.05) between adhesion of bacteria to the surfaces treated with rhamnolipids or surfactin and untreated control groups. Similarly, do Valle Gomes and Nitschke reported that adhesion of *Listeria monocytogenes* was reduced significantly after preconditioning of polystyrene surfaces with different concentrations of surfactin or rhamnolipids (38). According to previous reports by Jemil *et al.* (36) and Merghni *et al*. (24) analysis of cell adhesion reduction between different concentrations of rhamnolipids or surfactin confirmed that by raising biosurfactant concentration, the percentage of adhesion reduction increased. Van Hoogmoed et al. reported that biosurfactant derived from *Streptococcus mitis* BMS displayed inhibitory activities against two cariogenic bacteria (*S. mutans* ATCC 25175 and *Streptococcus sobrinus* HG 1025) adhesion to polystyrene wells in the presence or absence of pellicle (39). Surfactin and rhamnolipids are both anionic biosurfactants and negatively charged polystyrene surfaces after treatment; therefore, the anti-adhesive effect of biosurfactants could be related to the electrostatic repulsion between the negative charges of bacterial surface and polystyrene (38). Also, the pre-conditioning of polystyrene with surfactin or rhamnolipids resulted in surface hydrophobicity reduction due to biosurfactants orientation on the surface which reduced the hydrophobic interactions (40). 

*Disruption of pre-formed biofilm*

As shown in Tables 4 and 5, treatment of the polystyrene surfaces with 12.5 mg/mL of rhamnolipid dissociated about 67% of the *S. mutans* pre-formed biofilm, while 80 mg/mL of surfactin resulted in 62.2% of the *S. mutans* biofilm removal. There were significant differences among control group and 12.5 to 3.13 mg/mL of rhamnolipids and 80 to 40 mg/mL of surfactin (*P* < 0.05). We previously reported that Coryxin, a cyclic lipopeptide, produced by *Corynebacterium xerosis* NS5 showed adhesion inhibitory and disruptive effects against biofilm formation by a variety of bacteria (41). The reduction of bacterial adhesion and decreased biofilm population represent a clinically useful strategy in the removal of bacterial colonization from medical device surfaces, especially in UTIs. Velraeds *et al.* showed that the biosurfactant derived from *Lactobacillus acidophilus *inhibited the initial adhesion of uropathogenic *Enterococcus faecalis* on silicone rubber substrate and the growth of biofilm on human urine catheter (42-44). 

Our results indicated that higher concentrations of biosurfactants are needed for detachment of preformed biofilm from surface in comparison with their anti-adhesive activities. A surface active substance should penetrate into the biofilm and substrate interface in order to detach the biofilm. After their penetration, they could change the surface properties which leads to surface tension reduction and separation of biofilms from surfaces (45). 

In this study, rhamnolipids showed higher anti-adhesive and anti-biofilm activity against *S. mutans* compared with surfactin. Biosurfactants need to be adsorbed on the surfaces in order to change the surface tension; therefore, the dynamic of biosurfactant adsorption is an important factor in biosurfactant effectiveness. Biosurfactants have several hydrogen acceptor and donor groups in their molecules which form intramolecular hydrogen bonds. The remaining hydrogen acceptors and donors take part in interactions with surfaces. Hence, the biosurfactants which contain higher functional groups with hydrogen bonding potential, could condition the surfaces more effectively. Rhamnolipids contain more oxygen atoms in their functional groups than surfactin which explains higher ability of rhamnolipids in formation of intermolecular hydrogen bond in addition to their intramolecular bonds (38). 

**Table 1:** Reducing power of surfactin, rhamnolipids and vitamin C based on the equivalency to FeSO_4_

**Sample/antioxidant**	**Concentration** **(mM)**	**Equivalent to FeSO**_4_ **(μM)** ^*^
Surfactin	4.5 9	255.2 ± 3.1 1145.2 ± 1.8
Rhamnolipids	4.5 9	143.2 ± 2.9 537.2 ± 3.1
Vitamin C	0.35 0.7 1.4 2.8 5.6	22.8 ± 6.5 235.2 ± 3.7 553.2 ± 5.5 1415 ± 4 2500 ± 3.5

^*^Results were the averages of three replicates and represented as mean ± standard deviation.

**Table 2:** DPPH^a^ radical scavenging activity of surfactin and rhamnolipid biosurfactants in comparison to BHA^b^ and vitamin E

**Sample/Antioxidant**	**IC**_50 _ **(Concentration-activity correlation)**
Surfactin	2.73 mM (y = 10.299x + 22.293 , r^2 ^= 0.8976)
Rhamnolipids	4.15 mM (y = 3.8367x + 34.236 , r^2 ^= 0.9953)
Vitamin E	0.036 mM (y = 789.71x + 19.4 , r^2 ^= 0.9585)
BHA	2.86 mM (y = 16.057x + 4.4213 , r^2 ^= 0.9743)

^a^DPPH: 1, 1- diphenyl-2-picryl hydrazyl. ^b^BHA: butylated hydroxyanisole.

**Table 3:** Correlation between results of DPPH^a^ and FTC^b^ assay method for surfactin and rhamnolipid biosurfactants

**Biosurfactant**	**DPPH - FTC correlation**
Surfactin	y = 1.24x - 18.43 , r^2 ^= 0.859
Rhamnolipids	y = 0.763x + 16.21 , r^2 ^= 0.935

^a^DPPH: 1, 1- diphenyl-2-picryl hydrazyl. ^b^FTC: Ferric Thiocyanate.

**Table 4:** Removal percentages of one-day *Streptococcus mutans* biofilms formed on 96-well polystyrene plate surface and percent of adhesion inhibition of *S. mutans* with different concentrations of rhamnolipid solutions

**Biosurfactant concentration (mg/mL)**	**Anti-biofilm activity (%)**	**Anti-adhesive activity (%)**
12.5	66.7 ± 4.2^*^	93.7 ± 2.7
6.25	60.9 ± 7.8	91.5 ± 2.5
3.13	48.7 ± 5.5	90.8 ± 1.6
1.56	26.3 ± 4.3	92.2 ± 0.9
0.78	25.0 ± 5.8	78.7 ± 2.7
0.39	24.4 ± 4.4	70.1 ± 4.1
0.19	24.3 ± 5.6	63.1 ± 3.3
0.097	23.9 ± 6.1	51.2 ± 5.4

^*^Data are shown as mean of three independent replicates ± standard deviation.

**Table 5.** Removal percentages of one-day *Streptococcus mutans* biofilm formed on 96-well polystyrene plate surface and percent of adhesion inhibition of *S. mutans* with different concentrations of surfactin solutions

**Biosurfactant concentration (mg/mL)**	**Anti-biofilm activity (%)**	**Anti-adhesive activity (%)**
80	62.2 ± 7.1^*^	94.85 ± 1.47
40	53.7 ± .6.0	92.27 ± 1.27
20	46.3 ± 6.5	88.08 ± 1.9
10	35.8 ± 8.3	79.22 ± 3.95
5	23.9 ± 5.4	65.05 ± 4.10
2.5	21.9 ± 8.2	59.74 ± 3.62
1.25	23.4 ± 8.7	54.91 ± 3.62
0.63	22.9 ± 6.9	52.17 ± 5.57

^*^Data are shown as mean of three independent replicates ± standard deviation.

## Conclusion

In this study, antioxidant, lipid peroxidation inhibition, and anti-biofilm activities of rhamnolipids and surfactin were compared. Our results indicated that both rhamnolipid and glycolipid biosurfactants had antioxidant activity and lipid peroxidation inhibition capacity. Surfactin showed higher antioxidant activity than rhamnolipids and both biosurfactants seem to be less effective than common natural antioxidants including vitamin C and vitamin E. Surfactin showed relatively similar efficiency to BHA that suggests it as a good alternative for synthetic antioxidants. On the other hand, rhamnolipids could condition the surfaces more effectively and showed higher anti-adhesive and antibiofilm activity in comparison with surfactin. 

## References

[1] Xu Q, Nakajima M, Liu Z, Shiina T (2011). Biosurfactants for microbubble preparation and application. Int. J. Mol. Sci..

[2] Kiran GS, Priyadharsini S, Sajayan A, Priyadharsini GB, Poulose N, Selvin J (2017). Production of lipopeptide biosurfactant by a marine nesterenkonia sp. and its application in food industry.Front. Microbiol..

[3] Satpute SK, Zinjarde SS, Banat IM (2018). Recent updates on biosurfactants in the food industry. Microbial Cell Factories. 1st ed. CRC Press, Boca Raton.

[4] Nitschke M, Silva SSE (2018). Recent food applications of microbial surfactants. Crit. Rev. Food Sci. Nutr..

[5] Mnif I, Ghribi D (2016). Glycolipid biosurfactants: main properties and potential applications in agriculture and food industry. J. Sci. Food Agric..

[6] Nitschke M, Costa S, Mulligan CN, Sharma SK, Mudhoo A (2014). Biosurfactants in the food industry. Biosurfactants Research Trends and Applications.

[7] Desai JD, Banat IM (1997). Microbial production of surfactants and their commercial potential. Microbiol. Mol. Biol. Rev.

[8] Yalcin E, Cavusoglu K (2010). Structural analysis and antioxidant activity of a biosurfactant obtained from Bacillus subtilis RW-I. Turk. J. Biochem.

[9] Takahashi M, Morita T, Fukuoka T, Imura T, Kitamoto D (2012). Glycolipid biosurfactants, mannosylerythritol lipids, show antioxidant and protective effects against H2O2-induced oxidative stress in cultured human skin fibroblasts. J. Oleo. Sci.

[10] Valko M, Leibfritz D, Moncol J, Cronin MT, Mazur M, Telser J (2007). Free radicals and antioxidants in normal physiological functions and human disease. Int. J. Biochem. Cell Biol.

[11] Ohadi M, Forootanfar H, Rahimi HR, Jafari E, Shakibaie M, Eslaminejad T, Dehghannoudeh G (2017). Antioxidant potential and wound healing activity of biosurfactant produced by acinetobacter junii B6. Curr. Pharm. Biotechnol..

[12] Madhavi DL, Salunkhe DK (1995). Toxicological aspects of food antioxidants. Food Antioxidants: Technological, Toxicological and Health Perspectives.

[13] Yehye WA, Rahman NA, Ariffin A, Hamid SBA, Alhadi AA, Kadir FA, Yaeghoobi M (2015). Understanding the chemistry behind the antioxidant activities of butylated hydroxytoluene (BHT): A review. Eur. J. Med. Chem.

[14] Oteiza PI, Erlejman AG, Verstraeten SV, Keen CL, Fraga CG (2005). Flavonoid-membrane interactions: a protective role of flavonoids at the membrane surface? J. Immunol. Res.

[15] Ferreira A, Vecino X, Ferreira D, Cruz J, Moldes A, Rodrigues L (2017). Novel cosmetic formulations containing a biosurfactant from Lactobacillus paracasei. Colloids Surf. BBiointerfaces.

[16] Taghvaei M, Jafari SM (2015). Application and stability of natural antioxidants in edible oils in order to substitute synthetic additives. J. Food Sci. Technol..

[17] Høiby N, Bjarnsholt T, Givskov M, Molin S, Ciofu O (2010). Antibiotic resistance of bacterial biofilms. Int. J. Antimicrob. Agents.

[18] Colombo APV, Magalhaes CB, Hartenbach FaRR, Do Souto RM, Da Silva-Boghossian CM (2016). Periodontal-disease-associated biofilm: A reservoir for pathogens of medical importance. Microb. Pathog..

[19] Marsh P, Zaura E (2017). Dental biofilm: ecological interactions in health and disease. J. Clin. Periodontol.

[20] Akers KS, Cardile AP, Wenke JC, Clinton KM, Donelli G (2015). Biofilm formation by clinical isolates and its relevance to clinical infections. Biofilm-Based Healthcare-Associated Infections.

[21] Arciola CR, Campoccia D, Montanaro L (2018). Implant infections: adhesion, biofilm formation and immune evasion. Nat. Rev. Microbiol.

[22] Gbejuade HO, Lovering AM, Webb JC (2015). The role of microbial biofilms in prosthetic joint infections: a review. Acta Orthop.

[23] Meena KR, Kanwar SS (2015). Lipopeptides as the antifungal and antibacterial agents: applications in food safety and therapeutics. Biomed. Res. Int.

[24] Merghni A, Dallel I, Noumi E, Kadmi Y, Hentati H, Tobji S, Ben Amor A, Mastouri M (2017). Antioxidant and antiproliferative potential of biosurfactants isolated from Lactobacillus casei and their anti-biofilm effect in oral Staphylococcus aureus strains. Microb. Pathog.

[25] Samadi N, Abadian N, Ahmadkhaniha R, Amini F, Dalili D, Rastkari N, Safaripour E, Mohseni FA (Folia Microbiol (Praha)). Structural characterization and surface activities of biogenic rhamnolipid surfactants from Pseudomonas aeruginosa isolate MN1 and synergistic effects against methicillin-resistant Staphylococcus aureus.

[26] Linhardt RJ, Bakhit R, Daniels L, Mayerl F, Pickenhagen W (1989). Microbially produced rhamnolipid as a source of rhamnose. Biotechnol. Bioeng.

[27] Oyaizu M (1986). Studies on products of browning reaction: antioxidative activity of products of browning reaction. Jpn. J. Nutr. Diet.

[28] Mhadhebi L, Mhadhebi A, Robert J, Bouraoui A (2014). Antioxidant, anti-inflammatory and antiproliferative effects of aqueous extracts of three mediterranean brown seaweeds of the genus Cystoseira. Iran. J. Pharm. Res.

[29] Goodarzi S, Hadjiakhoondi A, Yassa N, Khanavi M, Tofighi Z (2016). Essential oils chemical composition, antioxidant activities and total phenols of Astrodaucus persicus. Iran. J. Basic. Med. Sci.

[30] Yassa N, Sharififar F, Shafiee A (2005). Otostegia persica as a source of natural antioxidants. Pharm. Biol.

[31] Kahlmeter G, Brown D, Goldstein F, Macgowan A, Mouton J, Odenholt I, Rodloff A, Soussyh CJ, Steinbakk M, Soriano F, Stetsiouk O (2006). European committee on antimicrobial susceptibility testing (EUCAST) technical notes on antimicrobial susceptibility testing. Clin. Microbiol. Infect.

[32] O’toole GA (2011). Microtiter dish biofilm formation assay. J. Vis. Exp.

[33] Yalcin E, Cavusoglu K (2010). Structural analysis and antioxidant activity of a biosurfactant obtained from Bacillus subtilis RW-I. Turk. J. Biochem.

[34] Tabbene O, Gharbi D, Slimene IB, Elkahoui S, Alfeddy MN, Cosette P, Mangoni ML, Jouenne T, Limam F (2012). Antioxidative and DNA protective effects of bacillomycin D-like lipopeptides produced by B38 strain. Appl. Biochem. Biotechnol.

[35] Ayed HB, Bardaa S, Moalla D, Jridi M, Maalej H, Sahnoun Z, Rebai T, Jacques P, Nasri M, Hmidet N (2015). Wound healing and in-vitro antioxidant activities of lipopeptides mixture produced by Bacillus mojavensis A21. Process. Biochem.

[36] Jemil N, Ayed HB, Manresa A, Nasri M, Hmidet N (2017). Antioxidant properties, antimicrobial and anti-adhesive activities of DCS1 lipopeptides from Bacillus methylotrophicus DCS1. BMC Microbiol.

[37] Tofani D, Balducci V, Gasperi T, Incerpi S, Gambacorta A (2010). Fatty acid hydroxytyrosyl esters: structure/antioxidant activity relationship by ABTS and in cell-culture DCF assays. J. Agric. Food Chem.

[38] Do Valle Gomes MZ, Nitschke M (2012). Evaluation of rhamnolipid and surfactin to reduce the adhesion and remove biofilms of individual and mixed cultures of food pathogenic bacteria. Food control.

[39] Van Hoogmoed CG, Van Der Mei HC, Busscher HJ (2004). The influence of biosurfactants released by S mitis BMS on the adhesion of pioneer strains and cariogenic bacteria. Biofouling.

[40] Shakerifard P, Gancel F, Jacques P, Faille C (2009). Effect of different Bacillus subtilis lipopeptides on surface hydrophobicity and adhesion of Bacillus cereus 98/4 spores to stainless steel and Teflon. Biofouling.

[41] Dalili D, Amini M, Faramarzi MA, Fazeli MR, Khoshayand MR, Samadi N (2015). Isolation and structural characterization of Coryxin, a novel cyclic lipopeptide from Corynebacterium xerosis NS5 having emulsifying and anti-biofilm activity. Colloids Surf. B Biointerfaces.

[42] Velraeds M, Van Der Mei H, Reid G, Busscher HJ (1996). Inhibition of initial adhesion of uropathogenic Enterococcus faecalis by biosurfactants from Lactobacillus isolates. Appl. Environ. Microbiol..

[43] Velraeds MM, Van De Belt-Gritter B, Busscher HJ, Reid G, Van Der Mei HC (2000). Inhibition of uropathogenic biofilm growth on silicone rubber in human urine by lactobacilli–a teleologic approach. World J. Urol..

[44] Velraeds MM, Van Der Mei HC, Reid G, Busscher HJ (1997). Inhibition of initial adhesion of uropathogenic Enterococcus faecalis to solid substrata by an adsorbed biosurfactant layer from Lactobacillus acidophilus. Urology.

[45] Mclandsborough L, Rodriguez A, Perez-Conesa D, Weiss J (2006). Biofilms: at the interface between biophysics and microbiology. Food Biophys.

